# Protective effect of cryotherapy against oral mucositis among allogeneic hematopoietic stem cell transplant recipients using melphalan-based conditioning

**DOI:** 10.1007/s00520-023-07989-9

**Published:** 2023-08-15

**Authors:** Saori Oku, Toshiko Futatsuki, Yoshiko Imamura, Haruna Hikita, Akemi Inada, Shinsuke Mizutani, Yasuo Mori, Haruhiko Kashiwazaki

**Affiliations:** 1 Section of Geriatric Dentistry and Perioperative Medicine in Dentistry, Division of Maxillofacial Diagnostic and Surgical Sciences, Faculty of Dental Science, Kyushu University, Fukuoka, Japan; 2 Hamanomachi Hospital, Fukuoka, Japan; 3 Dental Hygiene Section, Department of Medical Technology, Kyushu University Hospital, Kyushu University, Fukuoka, Japan; 4 Department of Nursing, Kyushu University Hospital, Kyushu University, Fukuoka, Japan; 5 OBT Research Center, Faculty of Dental Science, Kyushu University, 3-1-1 Maidashi, Higashi-Ku, Fukuoka, 812-8582 Japan; 6 Department of Medicine and Biosystemic Science, Kyushu University Graduate School of Medical Sciences, Fukuoka, Japan

**Keywords:** Oral mucositis, Oral cryotherapy, Methotrexate, Melphalan, Allogeneic hematopoietic stem cell transplantation, Binary logistic regression analysis

## Abstract

**Purpose:**

Oral cryotherapy is an effective method to prevent oral mucositis (OM) induced by chemotherapeutic agents, such as melphalan (Mel). However, there is limited data about cryotherapy in allogeneic hematopoietic stem cell transplantation (allo-HSCT) recipients; thus, the current study aimed to examine the efficacy of cryotherapy among allo-HSCT recipients treated with Mel-containing regimens.

**Methods:**

Medical records of 78 consecutive allo-HSCT recipients were retrospectively analyzed. Baseline characteristics and clinical courses between the patients who received cryotherapy (cryotherapy group, *n* = 42) and those who did not (control group, *n* = 36) were compared, especially focusing on methotrexate (MTX) use as a part of graft-versus-host disease (GVHD) prophylaxis.

**Results:**

Binary logistic regression analysis revealed that a higher dose of Mel (OR, 3.82; 95%CI, 1.085–13.46; *P* = 0.037) or MTX use (OR, 7.61; 95% CI, 2.41–23.97; *P* < 0.001) was associated with the incidence of OM. MTX use was also significantly associated with the duration of OM (β = 0.515; 95% CI, 9.712–21.636; *P* < 0.001). Among 31 patients without MTX use, cryotherapy was associated with a significant reduction of OM development (0% in the cryotherapy group vs 35% in the control group, *P* = 0.021). We did not find such an association in 47 patients with MTX use.

**Conclusion:**

Cryotherapy was useful to prevent the incidence of OM in allo-HSCT recipients in the cases without MTX for GVHD prophylaxis.

## Introduction

Allogeneic hematopoietic stem cell transplantation (allo-HSCT) is one the most powerful treatment options that can provide a cure for patients with hematological malignancies and inherited hematopoietic disorders [1, 2]. To ensure engraftment of infused donor cells, allo-HSCT recipients undergo intensive chemotherapy and/or total body irradiation (TBI), which eradicates not only residual malignant cells but also immunocompetent cells [2]. These procedures result in various complications in the oral cavity, including ulceration, gingivitis, infections, and oral mucositis (OM) [3], with the incidence of OM ranging from 60 to 100% [4, 5]. Regimen-related OM, which is dependent on the component drugs and intensity of conditioning regimens [6], may be associated with an impairment of systemic physical conditions (i.e., focal pain, malnutrition, increased risk of infection, decreased activities of daily living), change of treatment schedule [7], and prolonged hospitalization [8].

The reported risk factors for developing OM include high-dose melphalan (Mel), TBI, body mass index ≥ 25, and presence of methylenetetrahydrofolate reductase *677 TT* genotype, which is involved in folic acid metabolism [9]. In addition, the use of methotrexate (MTX), which is commonly administered to allo-HSCT patients as graft-versus-host disease (GVHD) prophylaxis, was recognized as another risk factor [10]. Therefore, folic acid (Leucovorin®) is sometimes initiated after MTX dose to reduce OM (known as leucovorin rescue) [11, 12], based on its reported efficacy [13].

To manage OM, palifermin (recombinant keratinocyte growth factor-1) and low-energy laser have been used [14–17]. In fact, palifermin treatment is the effective way to reduce the severity and the duration of OM for the hematologic cancer patients received intensive chemotherapy and radiotherapy. Low-energy laser is proved effective for the prevention of OM and the treatment of pain in the hematopoietic stem cell transplantation patients. In addition, a practice guideline from the Multinational Association of Supportive Care in Cancer/International Society of Oral Oncology (MASCC/ISOO) recommended cryotherapy for managing OM [18]. Cryotherapy, which involved cooling the buccal mucosa using ice, ice water, or ice cream, reduced the dispersion of chemotherapeutic agents into the circulation due to constriction of the blood vessels in the oral mucosa. Previous reports and a meta-analysis of randomized controlled trials have confirmed the efficacy of cryotherapy in preventing OM among patients who received high-dose Mel [19–22].

In this retrospective analysis, we explored the incidence and duration of OM among allo-HSCT recipients following Mel-containing preparative regimens as well as the impact of the concomitant use of MTX and prophylactic cryotherapy.

## Materials and Methods

### Study design, setting, and participants

This pre–post study recruited 93 patients who underwent allo-HSCT following Mel-containing conditioning regimens at Kyushu University hospital from April 2014 to December 2018. Among them, 12 patients with insufficient data and 3 patients who had undergone multiple allo-HSCT were excluded; thus, the remaining 78 patients were eligible for OM assessment in this study.

Cryotherapy was introduced at our institute in June 2018 to prevent Mel-induced OM. We compared the baseline characteristics and clinical courses between patients who underwent treatment, including prophylactic cryotherapy after June 2018 (cryotherapy group, *n* = 42) and those who did not prophylactic cryotherapy before May 2018 (control group, *n* = 36). We retrospectively reviewed their medical records and collected the following information: age, sex, underlying diseases, conditioning regimen, TBI, graft source, Mel dose, MTX use, leucovorin rescue, number of teeth, usage of denture, and incidence and duration of OM. The primary objective of this study was to evaluate the protective effect of cryotherapy for OM in allo-HSCT recipients following Mel-containing preparative regimens with or without short-term MTX. This study was approved by the Kyushu University Institutional Review Board for Clinical Research (approval number: 2021–417).

### Transplant procedures

All 78 patients received fludarabine/Mel-based conditioning regimens. In addition, patients either received 6.4–12.8 mg/kg of busulfan (*n* = 9), cytarabine (*n* = 18), cyclophosphamide (*n* = 1), anti-thymocyte globulin (*n* = 9), or 2 Gy (*n* = 38), 4 Gy (*n* = 18), and 12 Gy (*n* = 1) of TBI. Consequently, 5.13% (4/78) patients received myeloablative conditioning. Calcineurin inhibitors (cyclosporin or tacrolimus) were used as the backbone of GVHD prophylaxis; short-term MTX, mycophenolate mofetil, and methylprednisolone were administered in 47, 21, and 10 cases, respectively.

As bacterial prophylaxis, oral levofloxacin (500 mg/day) was generally administered from the beginning of the conditioning regimen to engraftment. Oral fluconazole or intravenous echinocandins (micafungin or caspofungin) and oral acyclovir were routinely given as prophylaxis for fungal infections and herpes simplex virus reactivation, respectively.

### Evaluation of OM and intervention

By using the National Cancer Institute Common Toxicity Criteria (NCI-CTCAE) version (v)　3.0 (Table 1), the status of the oral mucosa was assessed everyday by the nurses and at least once a week by three dentists from the beginning of the conditioning regimen until engraftment or OM resolution. The duration of OM was defined as the time taken to progress from Grade 1 to Grade 3 according to NCI-CTCAE v3.0. Conventional oral care, including low-energy laser, was administered by dentists as needed.

**Table 1:** Grading based on the National Cancer Institute Common Toxicity Criteria version 3.0

Oral mucositis (clinical exam)	
Grade 1	Erythema of the mucosa
Grade 2	Patchy ulcerations or pseudomembranes
Grade 3	Confluent ulcerations or pseudomembranes; bleeding with trauma
Grade 4	Tissue necrosis; significant spontaneous bleeding; life-threatening consequences

### Cryotherapy

Patients used ice cubes for cryotherapy. The ice cubes were contained in clean plastic cups. Patients could buy in the shop and stored in the freezer. Patients kept the ice cube in the mouth and spat the ice cube. They continued with this for a total of 90 min before and after Mel administration on 3 and 2 day before allo-HSCT.

### Statistical analysis

Age, number of teeth, and duration of oral mucositis were examined using the student’s t-test. Fisher’s exact test was used to analyze sex, TBI, Mel dose, MTX use, leucovorin rescue, denture usage, and OM incidence. Pearson’s Chi-square test was conducted to compare the underlying diseases and graft source.

Odds ratios (ORs) for the incidence of OM were calculated using a binary logistic regression analysis. We added age (continuous), sex (0: man, 1: woman), Mel dose (0: 80 mg/m^2^, 1: 140 mg/m^2^), MTX (0: no, 1: yes), TBI (0: no, 1: yes), cryotherapy (0: no, 1: yes), and leucovorin rescue (0: no, 1: yes) as independent variables for the binomial logistic regression analysis.

In addition, a multiple regression analysis was performed to identify the factors associated with the duration of OM; the independent variables used were age (continuous), sex (0: man, 1: woman), Mel dose (0: 80 mg/m^2^, 1: 140 mg/m^2^), MTX (0: no, 1: yes), TBI (0: no, 1: yes), cryotherapy (0: no, 1: yes) and leucovorin rescue (0: no, 1: yes).

Furthermore, we performed a subgroup analysis that focused on the concomitant use of MTX. This study analyzed the relationship between the incidence and duration of OM and the effectiveness of cryotherapy by dividing the patients into two groups based on the usage of Methotrexate.

All analyses were performed using the SPSS software program (version 28.0, IBM Japan, Tokyo). Statistical significance was defined as a *P* value < 0.05.

## Results

### Characteristics of participants

As shown in Table 2, there was no difference in the patient characteristics between the cryotherapy group (*n* = 42) and control group (*n* = 36), except for the use of TBI (85.7% vs 58.3%, *P* = 0.007). Neutrophil engraftment was 18.4 ± 5.4 days in the cryotherapy group and 18.8 ± 5.4 days in the control group (*P* = 0.756).

**Table 2:** Comparison of characteristics between cryotherapy and control groups

Variable		Cryotherapy (*n* = 42)	Control (*n* = 36)	*P* value
Age		54.8 ± 11.2 ^a^	53.2 ± 12.1	0.549 ^c^
Sex	Male	19 (45) ^b^	21 (58)	0.177^d^
Underlying disease	Acute myelogenous leukemia	15 (36)	11 (31)	0.381^e^
	Malignant lymphoma	11 (26)	11 (31)	
	Acute lymphoblastic leukemia	7 (17)	8 (22)	
	Myelodysplastic syndrome	4 (10)	1 (3)	
	Others	5 (12)	5 (14)	
Total body irradiation	No	6 (14)	15 (42)	0.007^d^
	Yes	36 (86)	21 (58)	
Graft source	Bone marrow	23 (55)	14 (39)	0.373^e^
	Peripheral blood	10 (24)	12 (33)	
	Cord blood	9 (21)	10 (28)	
Dose of melphalan	80 mg/m^2^	15 (36)	9 (25)	0.219 ^d^
	140 mg/m^2^	27 (64)	27 (75)	
Methotrexate	Yes	28 (67)	19 (53)	0.154^d^
	No	14 (33)	17 (47)	
Leucovorin rescue	Yes	7 (17)	3 (8)	0.326^d^
	No	35 (83)	33 (92)	
Neutrophil engraftment		18.4 ± 5.4	18.8 ± 5.4	0.756^c^
Number of teeth		22.7 ± 6.6	24.3 ± 7.2	0.328 ^c^
Usage of denture	Yes	11 (26)	4 (11)	0.080 ^d^

^a^ mean ± standard deviation, ^b^ n (%), ^c^ Student’s t-test, ^d^ Fisher’s exact test, ^e^ Pearson’s Chi-square test

### Incidence of OM and its risk factors

The incidence of OM was 47.6% (20/42) in the cryotherapy group and 55.6% (20/36) in the control group. In the cryotherapy group, the maximum grade of OM was 1 in 4.76% patients, 2 in 11.9%, 3 in 31.0%, and 4 in 0%. Whereas, in the control group, the maximum grade of OM was 1 in 8.33% patients, 2 in 19.4%, 3 in 27.8%, and 4 in 0%. We calculated ORs for the incidence of OM using binary logistic regression analysis and disclosed that high-dose Mel (140 mg/m^2^) (OR, 3.82; 95% CI, 1.09–13.46; *P* = 0.037) and MTX use (OR, 7.61; 95% CI, 2.41–23.97; *P* < 0.001) were statistically significantly correlated with OM development (Table 3). The protective effect of cryotherapy was not evident.

**Table 3:** Risk factors of the oral mucositis incidence as determined using binomial logistic regression analysis

Variables		Odds ratio	95% Confidence interval	*P-*value
Dose of Melphalan	80 mg/m^2^	1.000		
	140 mg/m^2^	3.820	1.085–13.455	0.037
Methotrexate	No	1.000		
	Yes	7.605	2.413–23.967	< 0.001

Model fit (forward selection (Likelihood ratio)): Hosmer–Lemeshow test (*P* = 0.946) and the accuracy of discrimination was 76.9%

Dependent variable: the incidence of oral mucositis (0: No, 1: Yes)

Independent variables: Age (continuous), sex (0: man, 1: woman), dose of Melphalan (0: 80 mg/m^2^, 1: 140 mg/m^2^), Methotrexate (0: no, 1: yes), total body irradiation (0: no, 1: yes), Cryotherapy (0: no, 1: yes) and Leucovorin rescue (0: no, 1: yes)

### Duration of OM and its risk factors

Next, we evaluated the duration of OM and its related factors. The median duration of OM was 7.0 days (range, 6.41–14.87 days) in the cryotherapy group and 7.0 days (range, 5.97–17.26 days) in the control group. Table 4 shows the risk factors of the duration of OM, as determined using the multiple regression analysis (*R*^2^ = 0.265). MTX use was significantly associated with the longer duration of OM (β = 0.515; 95%CI, 9.712–21.636; *P* < 0.001). Cryotherapy did not have impact on the duration of OM.

**Table 4:** Risk factors of the duration of oral mucositis as determined using multiple regression analysis

Variables	β	95% Confidence interval	*P-*value
Methotrexate	0.515	9.712–21.636	< 0.001

Dependent variable: the duration of oral mucositis (continuous)

Independent variables: Age (continuous), sex (0: man, 1: woman), dose of Melphalan (0: 80 mg/m^2^, 1: 140 mg/m^2^), Methotrexate (0: no, 1: yes), total body irradiation (0: no, 1: yes), Cryotherapy (0: no, 1: yes) and Leucovorin rescue (0: no, 1: yes)

### Subgroup analysis based on the MTX use

Since both Mel and MTX cause OM, and cryotherapy was adapted only for the duration of Mel administration, we further performed a subgroup analysis that focused on the concomitant use of MTX. Cryotherapy had a positive impact on OM prophylaxis in patients who were treated with Mel but not with MTX; no patient developed OM in the cryotherapy group (*n* = 14), whereas 6 out of 17 (35.3%) patients developed OM in the control group (*P* = 0.021) (Table 5). The cryotherapy group included a higher proportion of older patients with TBI use than the control group, indicating its robust efficacy in preventing OM in this cohort.

**Table 5:** Comparison of variables among those who received methotrexate and those who did not receive methotrexate in both the groups

Variable		No Methotrexate dose			Methotrexate dose		
		Cryotherapy (*n* = 14)	Control (*n* = 17)	*P* value	Cryotherapy (*n* = 28)	Control (*n* = 19)	*P* value
Age		57.9 ± 11.0^a^	49.3 ± 11.6^a^	0.046^c^	53.3 ± 11.1^a^	56.7 ± 11.7^a^	0.312^c^
Sex	Male	9 (64)^b^	7 (41)	0.200^e^	10 (27)^b^	14 (74)	0.011^e^
Underlying disease	Acute myelogenous leukemia	6 (43)	7 (41)	0.386^e^	9 (32)	4 (21)	0.314^e^
	Malignant lymphoma	5 (36)	3 (18)		6 (21)	8 (42)	
	Acute lymphoblastic leukemia	0 (0)	3 (18)		7 (25)	5 (26)	
	Myelodysplastic syndrome	1 (7)	1 (6)		3 (11)	0 (0)	
	Others	2 (14)	3 (18)		3 (11)	2 (11)	
Total body irradiation	None	1 (7)	9 (53)	0.008^d^	5 (18)	6 (32)	0.312^d^
	Yes	13 (93)	8 (47)		23 (82)	13 (68)	
Graft source	Bone marrow	2 (14)	1 (6)	0.591^e^	21 (75)	13 (68)	0.158^e^
	Peripheral blood	6 (43)	6 (35)		4 (14)	6 (32)	
	Cord blood	6 (43)	10 (59)		3 (11)	0 (0)	
Dose of Melphalan	80 mg/m^2^	9 (64)	8 (47)	0.337^d^	6 (21)	1 (5)	0.215^d^
	140 mg/m^2^	5 (36)	9 (53)		22 (79)	18 (95)	
Leucovorin rescue	Yes	-	-		7 (25)	3 (16)	0.718^d^
	No	-	-		21 (75)	16 (84)	
Oral mucositis	Yes	0 (0)	6 (35)	0.021^d^	20 (71)	14 (74)	0.865^e^
	No	14 (100)	11 (65)		8 (29)	5 (26)	
Days with oral mucositis (Grades 1 to 3)		-	8.5 ± 4.4		22.4. ± 11.0^a^	26.2 ± 18.5^a^	0.451^c^

^a^ mean ± standard deviation, ^b^ n (%), ^c^ Student’s t-test, ^d^ Fisher’s exact test, ^e^ Pearson’s Chi-square test

Among 47 patients who were treated with both Mel and MTX, 28 underwent prophylactic cryotherapy (Table 5). Leucovorin rescue was used for seven patients (25%) in the cryotherapy group and three patients (16%) in the control group (*P* = 0.718). The incidence of OM (20 of 28, 71% vs 14 of 19 74%, *P* = 0.865) and its duration (22.4 ± 11.0 days vs 26.2 ± 18.5 days, *P* = 0.451) were comparable between the two groups.

## Discussion

Cryotherapy is reportedly effective in preventing severe OM in patients treated with cytotoxic agents, especially high-dose Mel [23–26], in the setting of auto-HSCT. In addition, some previous studies have revealed that cryotherapy significantly reduced the incidence of all-grade OM [27] or moderate-to-severe OM [22, 28] even in allo-HSCT recipients. However, the efficacy of cryotherapy among allo-HSCT recipients was still controversial, presumably due to the variety of conditioning regimens, graft sources, and immunosuppressants used for GVHD prophylaxis. Therefore, in the current study, we assessed the impact of cryotherapy on OM development in 78 patients receiving Mel-based conditioning followed by the first allo-HSCT. Ice cubes were applied to the oral mucosa of the patients for a total of 90 min (30 min before, 30 min during administration, and 30 min after Mel administration); however, the most optimal timing and duration of oral cryotherapy remain unclear. A previous study revealed that 60-min cryotherapy had similar efficacy to 120-min cryotherapy [29]. Therefore, the best method of oral cryotherapy should be explored to prevent OM using different conditions (i.e., timing of cryotherapy and time and/or materials of cooling). Although various scales for the assessment of the severity of OM were available [30, 31], we adopted the NCI-CTCAE v3.0. instead of the latest v5.0. The latter allowed non-specialists of oral care to evaluate OM more easily by using pain and oral intake but not objectively about the degree of mucosal damage. To avoid the differences in proficiency among evaluators may affect the results, professional dentists with similar skill levels were engaged in this study. This was reconfirmed by performing daily assessment by non-specialists.

In this study, we aimed to determine whether cryotherapy was effective in patients with OM. First, we considered the risk factors that had an influence on the incidence and duration of OM. These analyses revealed that MTX was the risk factor for the incidence and duration of OM, and Mel was the risk factor for the incidence of OM. MTX, an inhibitor of folic acid metabolism, has reportedly altered the diversity and components of the gut and/or oral microbiota [32–34] and also caused mucositis similar to folic acid deficiency [35]. Another previous report demonstrated that MTX-containing GVHD prophylaxis resulted in more severe and prolonged OM than the non-MTX sirolimus-based method by inhibiting the repair of oral mucosa [36]. Therefore, we performed an additional analysis based on the MTX use to clarify the efficacy of cryotherapy in a more homogenous subgroup. Notably, the preventive effect of cryotherapy against OM development was evident in allo-HSCT recipients without MTX use. Regarding the patients who received short-term MTX, a shorter duration of OM was noted in the cryotherapy group than that in the control group; however, this difference was not significant.

Therefore, a future study should investigate the hypothesis that cryotherapy at the time of MTX administration contributes to the reduction and/or shortening of severe OM, although a prospective randomized study subjected to patients with myeloablative conditioning has failed [37]. Besides cryotherapy, alternative treatment strategies, including leucovorin rescue [12, 13], low-energy laser [38], and palifermin [15, 16], have emerged for managing OM. A combined use of these options with cryotherapy might succeed in reducing OM development even in patients with both Mel-based conditioning and MTX-containing GVHD prophylaxis.

There are several limitations in this study. First, it had a pre–post design; thus, patients analyzed in each group received allo-HSCT at different times. Although baseline characteristics were almost comparable between the two groups, differences may have existed in supportive care (e.g., antibiotics and antifungal agents). Second, our study was a single-center retrospective analysis with a small number of Japanese patients. We evaluated OM by using common scale NCI-CTCAE v3.0. However, this study was a retrospective study and we could not investigate the reliability and validity before the target period. Thus, the results should be interpreted cautiously. Third, this is an unavoidable problem for a study with a pre–post design, future studies should conduct randomized control trials to determine the effectiveness of cryotherapy for OM. At last, this study did not adequately assess the impact of cryotherapy on subjective conditions, including oral pain and quality of life-based patients’ satisfaction.

## Conclusion

This study revealed the usefulness of cryotherapy in preventing OM in allo-HSCT recipients without MTX use. Future randomized controlled studies with a larger number of patients are needed to confirm our findings.

## Data Availability

The data that support the findings of this study are available on request from the corresponding author, S.M.
